# Using structured reporting templates in staging pancreatic malignancies

**DOI:** 10.1186/1470-7330-15-S1-O7

**Published:** 2015-10-02

**Authors:** Isaac R Francis, Mahmoud M Al-Hawary, Ravi K Kaza

**Affiliations:** 1 Department of Radiology, University of Michigan Hospitals, Ann Arbor, Michigan 48109, USA

## 

Our clinical colleagues prefer structured or template reporting over conventional reports. The most commonly used type of conventional or free style reporting, tends to either “bury” the pertinent information needed for patient management in lengthy reports including unimportant incidental findings or fail to mention critical aspects of the findings that are crucial to the treatment and management of the current clinical problem. Structured or template reports on the other hand offer, the opportunity through the use of specific entry fields, to organize relevant information in an easily readable format and ensure completeness of the required information that is essential for patient management. For these reports to be practically helpful to the referring and treating physicians, they should be 1) concise, 2) use standardized terms and 3) easy to understand.

Several radiology societies and societies and organizations in other medical disciplines have started to provide examples of such templates to their members for use in clinical practice for ex. on their websites etc. (Radiological Society of North America, Society of Abdominal Radiology etc.). Usually the structured templates use standardized terms and avoid the use of ambiguous, vague and imprecise wording.

To take these reporting templates to the next level of being useful to the intended customer i.e. the referring medical specialist, it is essential that these reporting templates be developed in conjunction with physicians from the various disciplines that are involved in the care and management of the appropriate patient populations. This will ensure the use of mutually agreed upon terminology between the radiologist and the referring clinicians, eliminating any potential source of confusion.

For these reasons, we decided to formulate a working template for pancreatic adenocarcinoma, through a national effort. Working with the Society of Abdominal Radiology and the American Pancreatic Association, we put forth a consensus statement in 2014 which has been published simultaneously in two major journals: the American Journal of Gastroenterology and Radiology.

This workshop will illustrate the working template that we and others have developed with and are utilizing in patients with pancreatic adenocarcinoma using examples. We and others will be starting to work on developing a similar working reporting template with SAR as well gastroenterologists and surgeons for cystic pancreatic lesions later this year. Templates developed at other institutions will be shown and illustrated with examples.
